# Novel Magnetically Driven Superhydrophobic Sponges Coated with Asphaltene/Kaolin Nanoparticles for Effective Oil Spill Cleanup

**DOI:** 10.3390/nano12193527

**Published:** 2022-10-09

**Authors:** Qiang Chen, Lingling Zhang, Yuanhang Shan, Yindong Liu, Dongfeng Zhao

**Affiliations:** 1 State Key Laboratory of Heavy Oil Processing, College of Chemistry and Chemical Engineering, China University of Petroleum (East China), Qingdao 266580, China; 2 Key Laboratory of Coal Processing and Efficient Utilization (Ministry of Education), School of Chemical Engineering and Technology, China University of Mining and Technology, Xuzhou 221116, China; 3 Petrochemical Research Institute, PetroChina Co., Ltd., Beijing 100195, China

**Keywords:** asphaltene/kaolin/melamine nanocomposites, oil cleanup, absorption

## Abstract

Fast and effective cleanup of oil spills remains a global challenge. A modified commercial sponge with superhydrophobicity, strong absorption capacity, outstanding magnetic response, and fire resistance were fabricated by a facile and inexpensive route of dip-coated melamine sponge carbonization. The low-cost petroleum asphaltene and kaolin nanoparticles were used as the dip-coating reagent. High absorption capacity of the fabricated sponges allowed rapid and continuous removal of oil contaminants. Taking advantage of the good refractory property, the sponges can be used in burning conditions and directly reused after burning out of the absorbed oil. Reusability tests showed that the modified sponges still maintained high absorption capacity (>85%) after six regeneration and reuse cycles. These characteristics make the fabricated sponge a promising aid to promote effective in situ burning cleanup of oil spills, contributing as a magnetic oil collector and a fire-resistant flexible boom. An example usage scenario of the sponges applied to in situ burning cleanup of oil spills is described.

## 1. Introduction

With the rapid development of the petrochemical industry, oil spills have occurred more and more frequently and intensely in recent years [1,2,3,4]. The occurrence of oil spills, as a menace to the ecosystems, is a serious global environmental problem. The accidental leakages or deliberate release of petroleum hydrocarbons into marine environment leads to catastrophic damages to the ecological habitat and human health [5,6].

Oil slicks resulting from spills from ships, offshore oil platforms, as well as seafloor hydrocarbon reservoirs cause marine pollution [5,7]. Dong et al. [5] revealed that ocean oil slicks were dominantly contributed by anthropogenic discharges (94%) rather than natural seepages (6%). Unfortunately, elimination of anthropogenic discharges is not realistic given the demand of human development. A fast and effective cleanup method for oil spills is thus of particular importance for environmental protection.

Currently, common oil cleanup techniques can be categorized into four types: mechanical recovery [8,9], chemical methods [10,11], bioremediation [12,13], and in situ burning [14,15]. Gatou et al. [16] presented a summary on the benefits and drawbacks of each method. In brief, mechanical recovery using booms, barriers, or skimmers usually has a low cost but is of low selectivity and efficiency. Chemical dispersants can eliminate a large oil volume rapidly and effectively but bring secondary pollution. Bioremediation with microorganisms is cost effective and environmentally friendly, but usually shows slow degradation rates and low efficiency. In contrast, in situ burning offers a simple, fast, and inexpensive approach for removal of oil spills. Burning rapidly turns large quantities of oil into its primary gaseous combustion products (i.e., carbon dioxide and water), which eliminates the need for storage, transport, and disposal of recovered materials. Hence, in situ burning is recognized as one of the most promising techniques for rapid cleanup of large oil quantities at sea [15,17,18,19].

In situ oil spill burns require that the oil slick is thick enough (more than 1 mm [15]). Thus, spilled oil with dispersing thin layers cannot be burned directly. In addition, emulsion formation is detrimental to the oil burning; the presence of water-in-oil emulsions adds difficulty to ignite and once ignited reduces flame spreading. Therefore, this in situ burning method is not perfect. To expand its applicability and improve its performance, an advanced technique integrating multiple cleanup strategies needs to be developed [20,21,22].

Absorption is demonstrated to be an effective method to remediate oil pollution, considering that it is inexpensive, readily available, and environmentally friendly [23,24]. The key element for successful absorption is absorbent materials. Recently, polymeric sponges, such as melamine sponges, have been frequently used as a starting template for fabrication of oil absorption materials, given their outstanding features, such as highly porous three-dimensional structure, low cost, thermal stability, low bulk density, and excellent mechanical strength [25,26,27,28,29]. Nevertheless, the raw sponges have no oil/water selectivity because they are inherently both oleophilic and hydrophilic [25,30]. Therefore, various modification methods [31,32,33], including chemical grafting, coating, thermal treatment, etc., have been applied to alter the sponge wettability. Ruan et al. [31] fabricated a superhydrophobic sponge with excellent absorbency via immersions using dopamine and perfluorodecanethiol. By a two-step method, including spontaneous deposit of Fe_3_O_4_ nanoparticles and dip coating of lignin, Lei et al. [34] prepared multifunctional melamine sponges, which were hydrophobic, compressible, and magnetic responsive. However, the modifiers used in the above studies are expensive or unfit for large-scale applications. A cheap and scalable material remains to be developed.

Asphaltenes are defined operationally as the n-heptane insoluble, toluene soluble component of crude oil, which are a highly aromatic, polydisperse mixture consisting of the heaviest and most polar compounds in oil [35,36]. In the oil industry, asphaltenes cause severe problems, such as pipeline fouling and catalyst deactivation; and thus, they are usually treated as a “waste” material [36,37]. However, with the recent advances, novel value-added applications of asphaltenes in many fields (e.g., polymer industry, surface modification and interfaces) have been achieved by utilizing their unique multifunctional structures. Zhao et al. [38] and Yao et al. [39] fabricated asphaltene–melamine sponges by carbonization, showing high oil-absorption capability. Prior works [16,40,41] have demonstrated that asphaltenes contained abundant polycyclic aromatic hydrocarbons attached with various heteroatoms and thus could easily be adsorbed onto the sponge skeleton. Such adsorption could alter their wettability and, consequently, favor oil absorption.

In the present work, we developed a novel magnetic superhydrophobic asphaltene/kaolin/melamine sponge (m-AS/Kaolin@MS) with three aspects of improvements in comparison to the previously reported modified sponges: Firstly, the fabrication of low-cost asphaltene-modified melamine sponges with controlled movement under magnetic field; secondly, the novel introduction of kaolin nanoparticles in the composite sponges, which could simultaneously enhance the absorption capacity and magnetic response [42,43,44]; thirdly, an example usage scenario for the application of m-AS/Kaolin@MS to in situ burning of marine oil spills. Furthermore, the structure, wettability, oil absorbency, and flame resistance of the fabricated sponges were investigated.

## 2. Materials and Methods

### 2.1. Materials

Chemical substances including toluene, chloroform, n-hexane, ferric chloride, ethanol, hydrochloric acid (HCl), Sudan IV, methylene blue, and Tween 80 were purchased from Sinopharm Group Co., Ltd. (Shanghai, China). Kaolin was purchased from Shangxing Chem. Co., Ltd. (Maoming, China). Diesel was acquired from a local gas station (Qingdao, China). Peanut oil and melamine sponges were purchased from a local supermarket in Qingdao, China. Asphaltenes and crude oil were provided by an oil company in China.

### 2.2. Preparation of Samples

Preparation of Fe_3_O_4_@kaolin nanoparticles. Magnetic kaolin nanoparticles were synthesized by a chemical coprecipitation method. First, the raw kaolin sample was thermally treated at 250 °C for 2 h in a muffle furnace for purification and then mixed with a HCl (37%) solution to activate and expand the pore sizes of kaolin [45,46]. Typically, 10 mg of acid-treated kaolin powders were dispersed in 40 mL of deionized water under 10 min ultrasonication, followed by the addition of FeCl_2_·4H_2_O (0.3 mmol) and FeCl_3_ (0.6 mmol) in 60 mL H_2_O. After 24 h stirring, the product was separated by centrifugation and washed with water three times. The particles were calcined in a muffle furnace at 500 °C for 3 h to obtain the magnetic Fe_3_O_4_@Kaolin nanoparticles.

**Figure 1:**
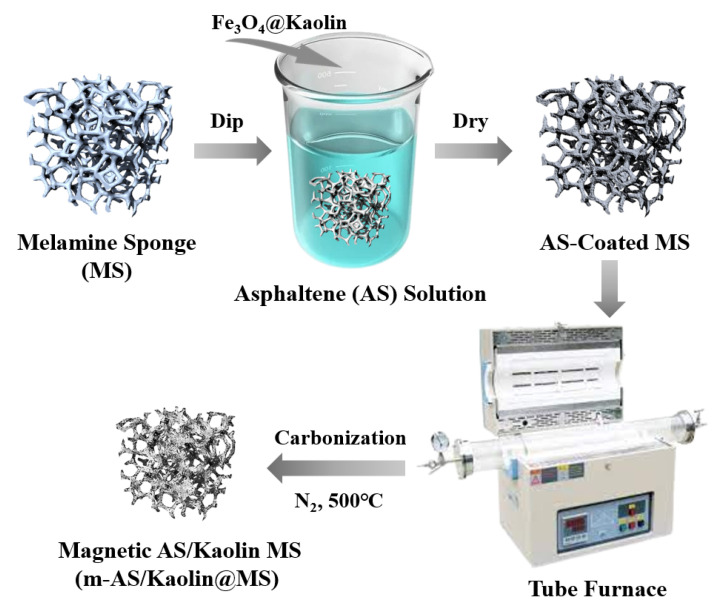
Schematic illustration of the fabrication process of the m-As/Kaolin@MS.

Preparation of superhydrophobic m-AS/Kaolin@MS. Melamine sponges were cleaned with deionized water and ethanol under ultrasonication for 20 min and dried at 105 °C. As schematically shown in Figure 1, the cleaned sponges were subsequently added to a 2-mg/mL asphaltene-in-toluene solution with 2-mg/mL magnetic kaolin nanoparticles. The above mixture was ultrasonically treated for 30 min followed by standing for 24 h. The asphaltene-coated melamine sponges (AS-coated MS) were collected, and excess solvent was removed by evaporation. The dried AS-coated MS was put in a tube furnace (YTH-2.5-10, TOLEDO, Shanghai, China) under N2 atmosphere and then heated up to 500 °C by 3 °C/min and maintained at 500 °C for 4 h to yield magnetic asphaltene/kaolin melamine sponges (m-AS/Kaolin@MS). High-temperature carbonization was used to stabilize and enhance the mechanical strength of the composite. For comparison, the asphaltene–melamine sponges (AS@MS) were also prepared following the same route as describe above but without the addition of Fe_3_O_4_@Kaolin particles.

### 2.3. Structural Characterization

The morphology and structures of the samples were examined with field-emission scanning electron microscopy (FESEM, GeminiSEM 500, Zeiss, Oberkochen, Germany). The weight and volume of the samples before and after carbonization were assessed using weighing balance and ruler. Thermogravimetric analysis (TGA) was performed on a Thermo Cahn TherMax 400 thermogravimetric analyzer (Thermo Electron, Waltham, MA, USA) by heating the samples at a rate of 10 °C/min to 1000 °C under an argon atmosphere. The data of weight were normalized by the weight of the sample at 100 °C to minimize the effects of moisture and gaseous species. The X-ray diffraction (XRD) analysis was carried out on a Rigaku Ultima IV diffractometer (Photonics Media, Pittsfield, MA, USA) operating at 30 mA and 40 kV.

### 2.4. Wettability and Absorption Tests

The wettability of the samples was first evaluated by direct observation using eye. Water and n-hexane oil were dyed by methylene blue and Sudan IV, respectively, which were then added dropwise onto the surface of the sponge samples to observe their visual appearance. The water/oil contact angles were measured using a drop shape analyzer. In a typical floating test, the sponge sample was put on the water surface and its floating condition was observed over time. A tweezer was used to push the floating sponge into water body. The images were captured by a Huawei Nova 7 phone camera.

A Sudan IV colored n-hexane oil droplet was placed on the surface of water. The oil-absorption processes of m-AS/Kaolin@MS were recorded to test its absorption capability. A Sudan IV colored chloroform oil, which is denser than water, was used to test the absorption capacity of the sponges at the subsurface of water. The sponges were forced into a shallow layer of water using a manet to contact with the chloroform droplet. Three chloroform droplets were absorbed continuously following a controlled sequence. In addition, a droplet of crude oil was used to observe the absorption behavior of the sponges with actual spilled oil.

The prepared sponges were immersed in 10 mL various oils (including hexane, chloroform, diesel, and peanut oil) for 5 min to reach saturation. Subsequently, the sponges were taken out and weighed to evaluate their absorption capacities (Cab) using the following equation

C_ab_ = (m − m_0_)/m_0_ (1)

where m is the weight of the sponge after oil absorption, and m0 is the weight of the sponge before oil absorption. After absorption saturation, the sample was then cleaned and compressed with a tweezer, followed by drying at 85 °C for 3 h. The regeneration and reuse cycle was repeated six times. The weights of the sponges were recorded before and after each cycle.

### 2.5. Demulsification and Burning Tests

An oil-in-water emulsion was prepared by mixing toluene and water (volume ratio of 1:20) using Tween 80 as emulsifier. A simple gravity-driven separating device using m-AS/Kaolin@MS as filter medium was employed to assess the oil–water separation performance. The sponges, both before and after oil absorption, were ignited by an alcohol lamp to test their refractory property.

## 3. Results and Discussion

### 3.1. Structural Features of the Asphaltene-Melamine Sponges

**Figure 2:**
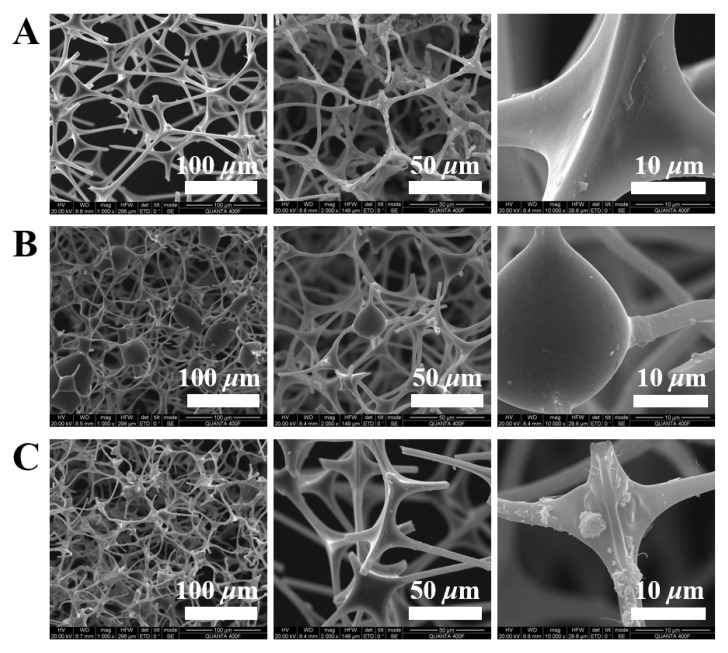
SEM images of MS (**A**), AS@MS (**B**), and m-AS/Kaolin@MS (**C**).

A commercial melamine sponge (MS) was used as a porous template, considering its high porosity and strong stability [47,48]. The structural morphology of the melamine sponges was investigated by SEM. Figure 2A–C shows the microstructures of the original melamine sponge (MS), the carbonized asphaltene-coated melamine (AS@MS), and the carbonized Fe_3_O_4_/asphaltene/kaolin-coated melamine (m-AS/Kaolin@MS), respectively. The MS illustrated an irregular three-dimensional interconnected network with abundant pores of sizes in the range of 50–100 μm. A higher magnification (Figure 2A, right) demonstrates a smooth and clean surface of the sponge skeleton.

The carbonization of asphaltene–melamine sponges did not significantly change the pore sizes of the sponge, but resulted in much more condensed networks (Figure 2B,C). The condensed porous structures are favorable for enhancing the adsorption capacity of crude oil. As shown in Figure 2B, a number of irregular bulge structures were observed at the joint parts of the AS@MS sample, most likely corresponding to the asphaltene-derived carbon materials. The addition of Fe_3_O_4_@Kaolin composites diminished the bulge features and thickened (thus strengthened) the sponge skeleton (Figure 2C), suggesting that the presence of these nanoparticles may improve the fluidity of asphaltenes during carbonization and thereby lead to an improved mutual integration of the asphaltene and melamine. A dispersed layer of nano-/micron-scale particles was firmly fixed on the sponge skeleton (Figure 2C, right), indicating the successful coating of magnetic particles.

As presented in Table 1, significant weight and volume losses were observed for both the AS@MS and m-AS/Kaolin@MS during the carbonization process, which is consistent with the SEM results showing condensed pore structures (Figure 2B,C). In comparison to the AS@MS, the less weight and volume loss of the m-AS/Kaolin@MS is largely due to the presence of thermally stable Fe_3_O_4_@Kaolin particles, further suggesting their important role to the structural development of asphaltene–melamine sponge during carbonization.

**Figure 3:**
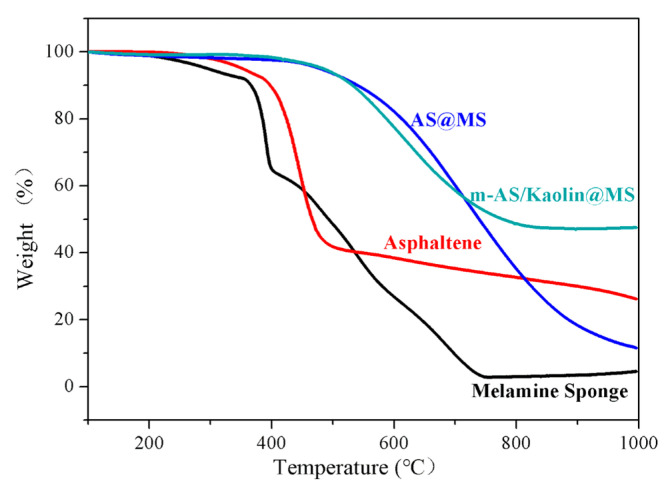
Thermogravimetric analysis (TGA) curves of the melamine sponge, asphaltene, AS@MS, and m-AS/Kaolin@MS samples.

In addition, thermal stabilities of the samples were investigated by thermogravimetric analysis. TGA curves of the MS, asphaltene, AS@MS, and m-AS/Kaolin@MS are shown in Figure 3. The weight of pure MS is stable before 350 °C, confirming the stability of the sponges. A rapid weight loss in MS was observed from 370 to 750 °C (Figure 3). The extreme weight loss around 400 °C is attributed to the breakdown of methylene bridges, and weight losses in temperature higher than 400 °C are largely caused by the thermal decomposition of the triazine [49,50]. The thermal decomposition of the asphaltene mainly occurred in the temperature range of 350 to 490 °C, which is consistent with the thermal behavior of the reported typical asphaltenes [51,52]. No significant weight loss was observed in the AS@MS and m-AS/Kaolin@MS before 500 °C, due to the pre-carbonization processes. Among the investigated samples, the highest residue weight of m-AS/Kaolin@MS after the TGA tests suggests its strong thermal stability, which is consistent with the results in Table 1.

**Figure 4:**
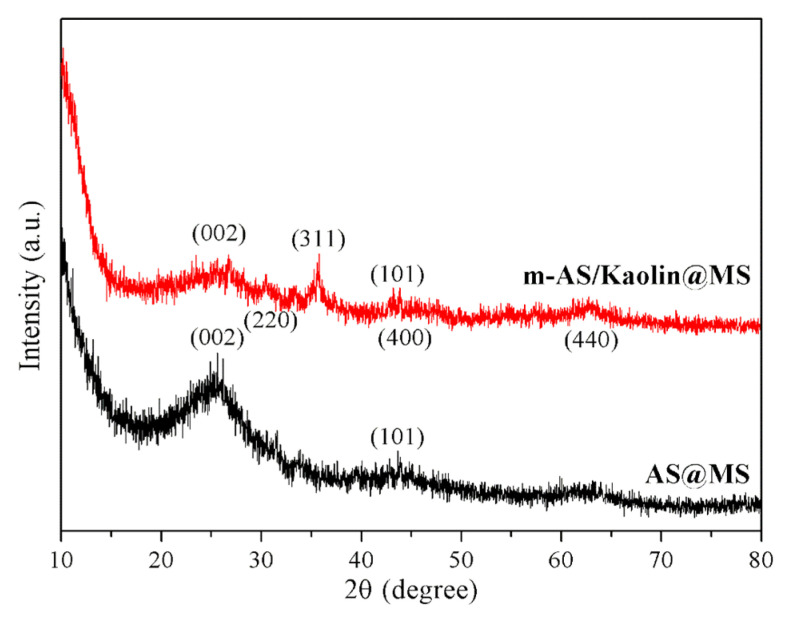
XRD diffractograms of the AS@MS and m-AS/Kaolin@MS.

The structure of the prepared sponges was further characterized by XRD. The diffractograms of the AS@MS and m-AS/Kaolin@MS in the 2θ interval ranging from 10 to 80° are shown in Figure 4. Both samples presented a characteristic peak at 2θ angle of 26° as well as a very weak signal at 43°, corresponding to the (002) and (101) diffraction planes in the graphitic nanocrystalline structure, respectively. Additionally, the m-AS/Kaolin@MS sample had peaks at 2θ angle of 30.5, 35.5, 43.5, and 62.5°, which correspond to the characteristic peaks of Fe_3_O_4_. The results confirm the successful adsorption of carbonaceous asphaltene and magnetic particles onto the sponge skeleton.

### 3.2. Wettability Characterization

**Figure 5:**
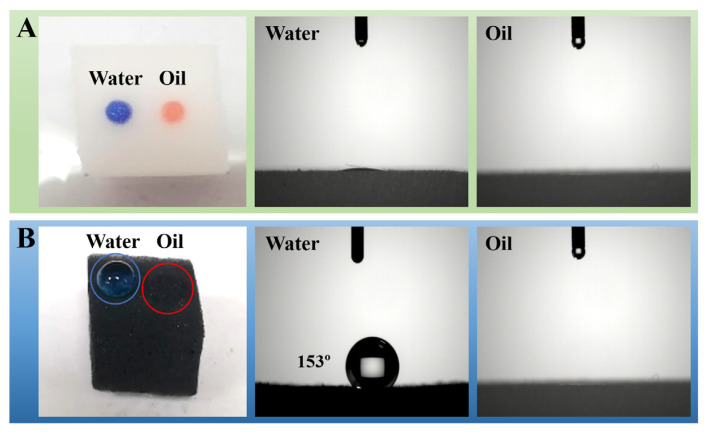
Visual appearance and contact angles of a water/oil droplet on an untreated melamine sponge (**A**) and a m-AS/Kaolin@MS (**B**). The water droplet was colored by methylene blue, and the n-hexane oil droplet was colored by Sudan IV.

The wettability of the pure sponge and modified sponge was evaluated by visual water/oil droplet tests and contact angle measurements. As shown in Figure 5A, the colored water and oil droplets were rapidly absorbed into the pure sponge, with both the water and oil contact angles of 0°. The observations indicate that the untreated commercial sponges can absorb both water and oil with no selectivity. In contrast, the water drop attained spherical shape when placed on the surface of the m-AS/Kaolin@MS, with a water contact angle of 153°, while the oil droplet was quickly absorbed giving an oil contact angle of 0° (Figure 5B). The results demonstrate the superhydrophobic property of the prepared m-AS/Kaolin@MS sample. The high hydrophobicity is attributed to the asphaltene coating on the melamine sponge skeletons.

**Figure 6:**
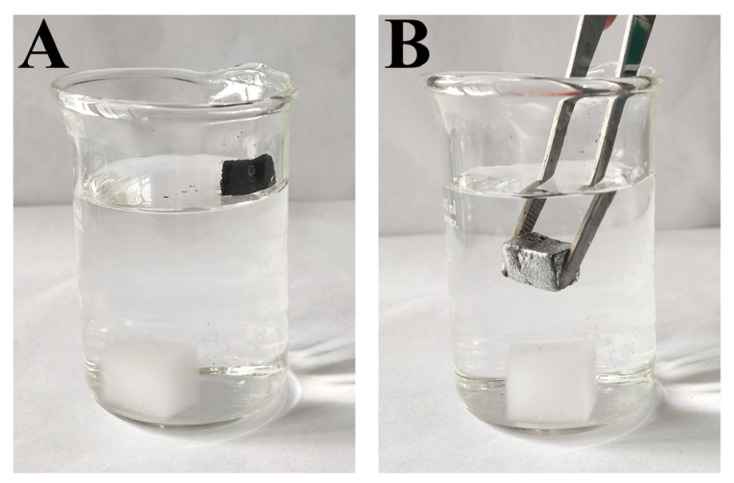
Floating tests of the untreated MS and m-AS/Kaolin@MS on the water surface. Natural appearance after 5 min (**A**). Forcing the m-AS/Kaolin@MS sample to immerse into the water by using a tweezer (**B**).

When placed on the water surface, the pure sponge sank to bottom of the beaker within 5 min due to the absorption of water, while the asphaltene-modified sponge kept floating on the water surface (Figure 6A). The floating capability of the m-AS/Kaolin@MS is favorable for its use in cleanup of oil spills on sea surface. As shown in Figure 6B, a number of bubbles were observed all over the m-AS/Kaolin@MS sample when it was immersed into the water, an observation consistent with its superhydrophobicity (Figure 6B).

### 3.3. Absorption Capability

**Figure 7:**
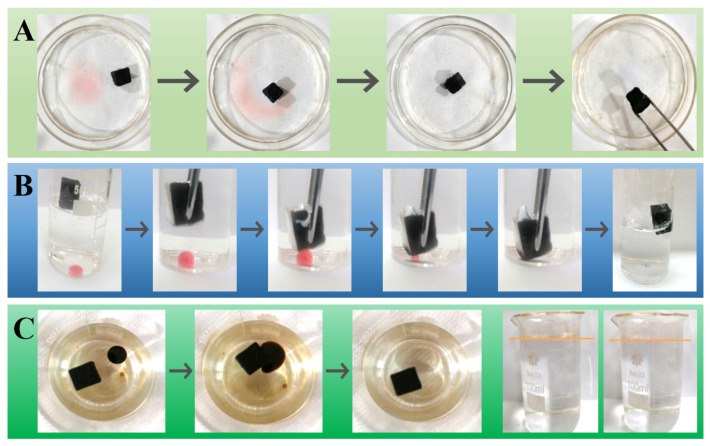
Removing oil from the top surface (**A**,**C**) and subsurface (**B**) of water using the m-AS/Kaolin@MS. The oil contaminants were the n-hexane ((**A**), colored by Sudan IV), chloroform ((**B**), colored by Sudan IV), and crude oil (**C**).

The absorption capability of the modified sponge was assessed from multiple aspects. Figure 7A shows the absorption process of m-AS/Kaolin@MS for an oil film on the top surface of water. When the modified sponge was placed in the water, the n-hexane oil was rapidly and completely absorbed into the sponge, illustrating an efficient cleanup of oil contaminant on the water surface. The absorbed oil was retained in the m-AS/Kaolin@MS with no leak when it was taken out from the water surface (Figure 7A, right), indicating its good oil retention capacity.

To investigate the absorption performance in underwater conditions, an oil droplet (oily red dyed chloroform) was dropped into the water and subsequently sank to the bottom (Figure 7B). A tweezer was used to force the modified sponge into the subsurface of water, making contact with the oil droplet at the bottom. It was observed that the oil droplet can be completely absorbed into the sponge after making contact. When releasing the force, the sponge with absorbed oil immediately and spontaneously floated to the water surface (Figure 7B, right). This observation demonstrates the underwater absorption capability of the prepared sponges.

The absorption capability of the sponge was further verified using high-viscosity crude oil (Figure 7C). As expected, when the m-AS/Kaolin@MS sample came in contact with crude oil on the surface of water, the oil was quickly absorbed into the sponge, and it remained floating after the absorption was completed (Figure 7C). No significant change was observed to the volume of water before and after crude oil cleanup, further confirming its high absorption selectivity of oil against water (Figure 5 and Figure 6).

**Figure 8:**
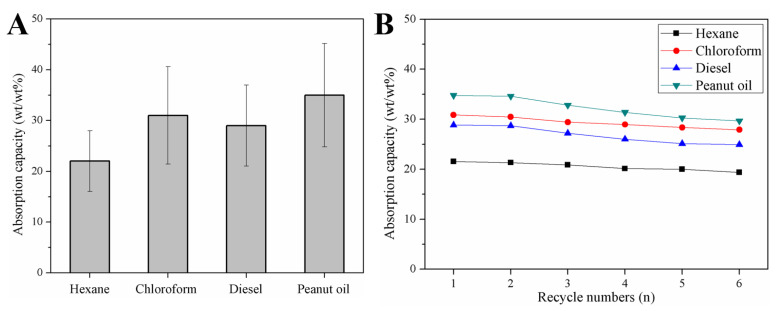
Absorption capacities (**A**) and recyclability (**B**) of the m-AS/Kaolin@MS.

The absorption capacities of the modified sponge for various oils (i.e., hexane, ethanol, chloroform, diesel, and peanut oil) were quantitatively determined based on the weight at absorption saturation using Equation 1. As shown in Figure 8A, the modified sponge was able to absorb a variety of oils with high absorption capacities. However, the observed absorption values were lower than the values reported by Ruan et al. [31] and Yang et al. [53], likely due to the presence of magnetic Fe_3_O_4_ particles and the carbonization operation at 500 °C. The multifunctional features of the prepared sponges largely expand their usage in various practical applications, although with the sacrifice of reduced absorption capacity. After six recycles, the absorption capacity still remains more than 85% of the initial values, suggesting the excellent reusability of the m-AS/Kaolin@MS (Figure 8B).

**Figure 9:**
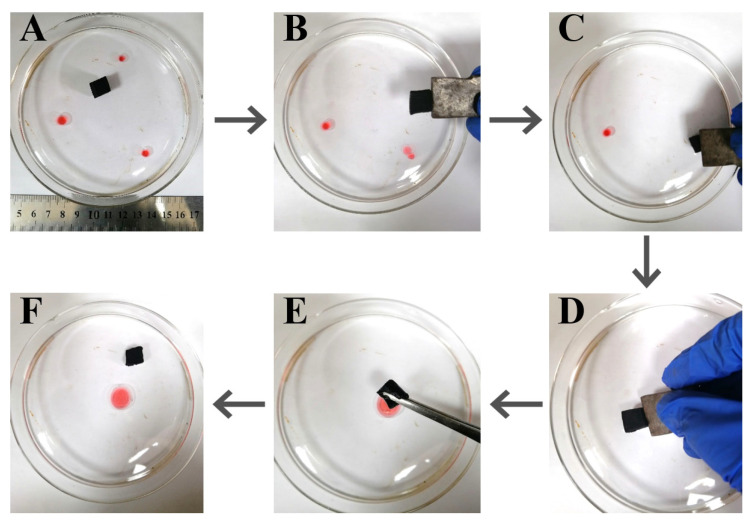
Magnetically guided cleanup of three chloroform droplets from water by using the m-AS/Kaolin@MS: On the way to the first droplet (**A**); cleaning the first droplet (**B**); cleaning the second droplet (**C**); cleaning the third droplet (**D**); squeezing out the oil using a tweezer (**E**); and recovered oil and recycled sponge (**F**).

Another important feature of our prepared sponges is their magnetic response; the modified sponges can be precisely navigated and anchored to the oil-contaminated sites to cleanup. Figure 9 demonstrates the cleaning process of three chloroform droplets from water. By controlling the orientation of the magnet, the sponge was rapidly and precisely guided to the oil droplets. Upon contact with the targeted oil droplet, the sponge was kept in place for seconds, and the oil can be quickly absorbed. After cleanup of one droplet, the sponge was directly driven to the next droplet to operate (Figure 9A–D). The water phase appeared to be very clean without any visible oil residue after continuous absorption of the three droplets, reflecting the high absorption capacity and removal efficiency. The absorbed oil can be easily squeezed out by using a tweezer (Figure 9E) and the sponge can recover to the original shape once removing the tweezer (Figure 9F). The observations indicate the superior compressibility and recyclability of the modified sponge.

### 3.4. Oil–Water Demulsification

**Figure 10:**
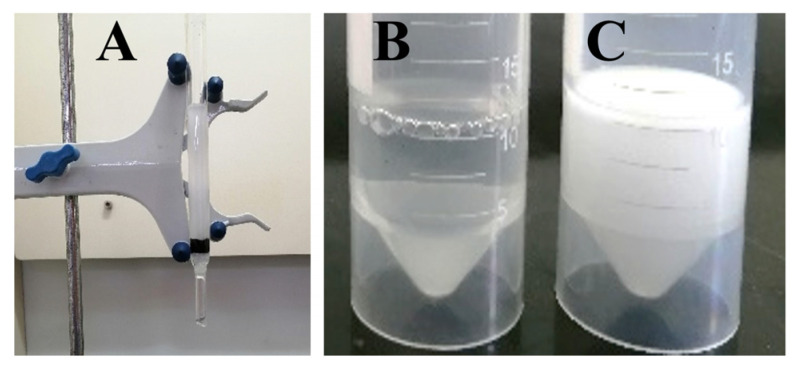
Demulsification using the m-AS/Kaolin@MS as filter medium: the experimental setup (**A**), the obtained filtrate after filtration (**B**), and the original emulsion before filtration (**C**).

The presence of oil–water emulsions adds to the difficulty of cleanup of oil spills [54,55]. The demulsification property of the modified sponge was studied using a simple device as shown in Figure 10A. The liquid changed from turbid emulsions (Figure 10C) to transparent water (Figure 10B) by simply passing through the m-AS/Kaolin@MS under gravity, indicating the good demulsification capability of the modified sponge. This paves the way for its application in emulsified water cleanup.

### 3.5. Refractory Property

**Figure 11:**
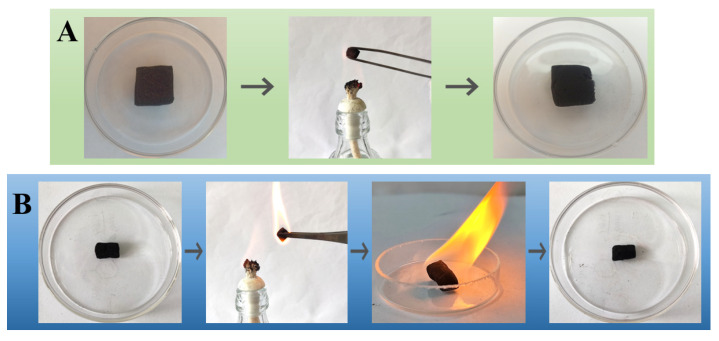
Burning of the m-AS/Kaolin@MS before (**A**) and after (**B**) absorption of crude oil.

The refractory property of the modified sponge under burning conditions is investigated. As shown in Figure 11A, the m-AS/Kaolin@MS (without any absorbed oil) cannot be ignited by the alcohol lamp flame. No significant change was observed in the shape or size of the sponge sample under the burning flame (Figure 11A), illustrating its good fire resistance performance. Figure 11B corresponds to the m-AS/Kaolin@MS sample with absorbed crude oil. The sample was easily ignited by the lamp flame. A flaming fire was observed due to the presence of crude oil (Figure 11B, middle), showing a complete combustion with no smoke. The fire ended naturally after a few minutes when the crude oil was burned up. The m-AS/Kaolin@MS still retained its original shape and size after burning (Figure 11B, right), further demonstrating its good refractory property. The sponges after burning retained good absorption capability, which can be directly re-used for further absorption operations.

### 3.6. Application Scenario in In Situ Burning of Oil Spills

Considering the excellent performances demonstrated in the above tests, our modified sponge offers new solutions to tackle oil contamination under various environments, for instance, in situ burning of oil spills at sea. An example application scenario is described as follows; An oil spill occurs, either accidentally or deliberately, in a marine environment. The sponges (m-AS/Kaolin@MS) are dropped to target the area. The sizes of the sponges can be tailored and adjusted according to the requirement of actual application. Owing to their high absorption capacity (Figure 7, Figure 8 and Figure 9), the spilled oil is expected to be demulsified and absorbed, effectively and rapidly (Figure 7, Figure 8 and Figure 10). The sponges with adsorbed oil are magnetically driven following a desired route, resulting in a simultaneous collection of the dispersed oil to give thick oil slicks. Thus, by forming a flexible and fire-resistant boom, the distributed oil spills are well collected for an efficient in situ burning. The burning process can remove the absorbed oil in the sponges and thus, no additional recycling operations are needed. Overall, in situ burning with the aid of the magnetically driven superhydrophobic sponges provides a fast, facile, and effective strategy for cleanup of oil spills.

## 4. Conclusions

A superhydrophobic asphaltene/kaolin/melamine sponge with magnetic response and fire resistance properties was prepared via mild carbonization of asphaltene-coated sponges. A value-added use of low-cost asphaltene and kaolin for high-performance sponges was achieved. The as-acquired product exhibited opening interconnected porous structures, high water repellence (water contact angle of 153°), excellent oil absorption performance (highly selective absorption of oil against water), flexible compressibility, good recyclability and reusability, and good refractory and thermal stability. The sizes of the sponges can be tailored depending on the application scenario, and the fabrication of this sponge is easy to scale up. Taking advantage of these characteristics, we believe that this high-performance sponge can be widely used to tackle oil contamination in various environments, such as in situ burning cleanup of marine oil spills.

## Figures and Tables

**Table 1:** Weight loss of the AS@MS and m-AS/Kaolin@MS samples during the carbonization at 500 °C for 4 h.

	AS@MS	m-AS/Kaolin@MS
weight loss (wt%)	82	67
volume loss (vol%)	86	78

## Data Availability

Not applicable.
